# Effect of Plyometric Jump Training on Skeletal Muscle Hypertrophy in Healthy Individuals: A Systematic Review With Multilevel Meta-Analysis

**DOI:** 10.3389/fphys.2022.888464

**Published:** 2022-06-27

**Authors:** F. Arntz, B. Mkaouer, A. Markov, B. J. Schoenfeld, J. Moran, R. Ramirez-Campillo, M. Behrens, P. Baumert, R. M. Erskine, L. Hauser, H. Chaabene

**Affiliations:** ^1^ Division of Training and Movement Sciences, Research Focus Cognition Sciences, University of Potsdam, Potsdam, Germany; ^2^ Department of Individual Sports, Higher Institute of Sport and Physical Education of Ksar Said, University of Manouba, Tunis, Tunisia; ^3^ Department of Health Sciences, CUNY Lehman College, Bronx, NY, United States; ^4^ Rehabilitation and Exercise Sciences, School of Sport, University of Essex, Colchester, United Kingdom; ^5^ Department of Physical Activity Sciences, Universidad de Los Lagos, Osorno, Chile; ^6^ Exercise and Rehabilitation Sciences Laboratory, Faculty of Rehabilitation Sciences, School of Physical Therapy, Universidad Andres Bello, Santiago, Chile; ^7^ Department of Sport Science, Institute III, Otto-von-Guericke University Magdeburg, Magdeburg, Germany; ^8^ Department of Orthopedics, University Medicine Rostock, Rostock, Germany; ^9^ Exercise Biology Group, Faculty of Sport and Health Sciences, Technical University of Munich, Munich, Germany; ^10^ School of Sport and Exercise Sciences, Liverpool John Moores University, Liverpool, United Kingdom; ^11^ Institute of Sport, Exercise and Health, University College London, London, United Kingdom; ^12^ Department of Sports and Health Sciences, Faculty of Human Sciences, University of Potsdam, Potsdam, Germany

**Keywords:** muscle tissue, muscle strength, stretch shortening cycle exercise, muscle growth, human physical conditioning, youth sports, aged

## Abstract

**Objective:** To examine the effect of plyometric jump training on skeletal muscle hypertrophy in healthy individuals.

**Methods:** A systematic literature search was conducted in the databases PubMed, SPORTDiscus, Web of Science, and Cochrane Library up to September 2021.

**Results:** Fifteen studies met the inclusion criteria. The main overall finding (44 effect sizes across 15 clusters median = 2, range = 1–15 effects per cluster) indicated that plyometric jump training had small to moderate effects [standardised mean difference (SMD) = 0.47 (95% CIs = 0.23–0.71); *p* < 0.001] on skeletal muscle hypertrophy. Subgroup analyses for training experience revealed trivial to large effects in non-athletes [SMD = 0.55 (95% CIs = 0.18–0.93); *p* = 0.007] and trivial to moderate effects in athletes [SMD = 0.33 (95% CIs = 0.16–0.51); *p* = 0.001]. Regarding muscle groups, results showed moderate effects for the knee extensors [SMD = 0.72 (95% CIs = 0.66–0.78), *p* < 0.001] and equivocal effects for the plantar flexors [SMD = 0.65 (95% CIs = −0.25–1.55); *p* = 0.143]. As to the assessment methods of skeletal muscle hypertrophy, findings indicated trivial to small effects for prediction equations [SMD = 0.29 (95% CIs = 0.16–0.42); *p* < 0.001] and moderate-to-large effects for ultrasound imaging [SMD = 0.74 (95% CIs = 0.59–0.89); *p* < 0.001]. Meta-regression analysis indicated that the weekly session frequency moderates the effect of plyometric jump training on skeletal muscle hypertrophy, with a higher weekly session frequency inducing larger hypertrophic gains [β = 0.3233 (95% CIs = 0.2041–0.4425); *p* < 0.001]. We found no clear evidence that age, sex, total training period, single session duration, or the number of jumps per week moderate the effect of plyometric jump training on skeletal muscle hypertrophy [β = −0.0133 to 0.0433 (95% CIs = −0.0387 to 0.1215); *p* = 0.101–0.751].

**Conclusion:** Plyometric jump training can induce skeletal muscle hypertrophy, regardless of age and sex. There is evidence for relatively larger effects in non-athletes compared with athletes. Further, the weekly session frequency seems to moderate the effect of plyometric jump training on skeletal muscle hypertrophy, whereby more frequent weekly plyometric jump training sessions elicit larger hypertrophic adaptations.

## 1 Introduction

Plyometric jump training is a popular form of physical conditioning in both general (Moran et al., 2018; Izquierdo et al., 2021) and athletic populations (Ramirez-Campillo et al., 2018; Ramirez-Campillo et al., 2020b). It constitutes a suitable training option in a resource-constrained setting as it can be carried out without the need for equipment. Plyometric exercises involve the physiological phenomenon called the “stretch-shortening cycle” (Ishikawa and Komi, 2008; Taube et al., 2012), which consists of a rapid eccentric action of the agonistic muscle-tendon unit immediately followed by rapid concentric action of that same muscle-tendon unit (Komi, 1984; Taube et al., 2012). The main advantage of the stretch-shortening cycle compared with isolated concentric or eccentric muscle actions is the storage and subsequent release of kinetic energy eliciting greater power production (Dietz et al., 1979; Voigt et al., 1998). Generally, the efficiency of the stretch-shortening cycle is underpinned by a complex interaction of multiple hierarchical levels of the central nervous system including the coordination of anticipated (feedforward) and reflex (feedback) mechanisms [for more insights see Taube et al. (2012)].

There is persuasive evidence on the effectiveness of plyometric jump training on a wide range of measures of physical fitness (e.g., muscle strength, muscle power, sprint speed, and balance) regardless of age, sex, and training experience (Bedoya et al., 2015; Ramírez-Campillo et al., 2015; Chaabene and Negra, 2017; Moran et al., 2018; Chaabene et al., 2019; Vetrovsky et al., 2019; Ramirez-Campillo et al., 2020a; Ramirez-Campillo et al., 2021a; Ramirez-Campillo et al., 2021b). Additionally, plyometric jump training benefits many parameters of health (e.g., bone mineral density, injury prevention, and fall prevention) (Markovic and Mikulic, 2010; Kish et al., 2015; Gómez-Bruton et al., 2017; Vlachopoulos et al., 2018; Vetrovsky et al., 2019; Bull et al., 2020; Huang et al., 2020). The benefits of plyometric jump training are mainly attributable to an increased neural drive to the active muscles (Häkkinen et al., 1985; Häkkinen et al., 1990; Chimera et al., 2004; Kyröläinen et al., 2005; Fouré et al., 2012; Suchomel et al., 2018). More specifically, the increase in muscle strength and power following plyometric jump training has usually been attributed to increased neuromuscular activation (e.g., motor unit recruitment, firing frequency, synchronization, etc.) and better inter-muscular coordination (e.g., decreased co-activation of the antagonist).

Unlike traditional resistance training, the effects of plyometric jump training on skeletal muscle hypertrophy have received little attention in the literature. Skeletal muscle hypertrophy can be defined as an increase in muscle mass and cross-sectional area (CSA) at the level of the entire muscle as well as individual muscle fibers (Russell et al., 2000; Schoenfeld et al., 2021). It can be directly measured using macroscopic [e.g., B-mode ultrasound, magnetic resonance imaging (MRI)] or microscopic (e.g., biopsy) assessment methods (Haun et al., 2019). Additionally, an indirect method has also been developed and applied using a prediction equation to assess muscle volume and CSA (Chelly et al., 2006). Of note, the effects of plyometric jump training on skeletal muscle hypertrophy seem to be equivocal in the existing literature. For instance, Kyröläinen et al. (2005) examined the effects of plyometric jump training on fiber CSA of the lateral gastrocnemius muscle in recreationally active males aged 24 years and reported no changes after 15 weeks of training. Likewise, Fouré et al. (2012) studied the effects of 14 weeks of plyometric jump training on the CSA of the gastrocnemius muscles in healthy active males aged 20 years and revealed no significant changes after training. In contrast, Kubo et al. (2007) reported a significant increase in plantar flexor muscle volume (∼5%) following 12 weeks of plyometric jump training in healthy untrained males aged 22 years. Additionally, Malisoux et al. (2006b) examined the effects of 8 weeks of plyometric jump training on single fiber CSA in recreationally active males aged 23 years. These authors revealed a significant increase in the CSA of type I (+23%), type IIa (+22%), and type IIa/IIx fibers (+30%) in the vastus lateralis muscle. Markovic and Mikulic (2010) conducted a comprehensive review of the effects of plyometric jump training on neuromuscular and performance outcomes and concluded that plyometric jump training has the potential to enhance skeletal muscle hypertrophy but to a lesser extent compared with traditional resistance training. Recently, Ramírez-delaCruz et al. (2022) conducted a systematic review and meta-analysis on the effects of plyometric jump training on lower body muscle architecture in healthy adults (≥18 years). The authors revealed that plyometric jump training is an effective method to increase muscle thickness of the vastus lateralis, vastus medialis, rectus femoris, and triceps surae. They additionally concluded that plyometric jump training is effective in increasing fascicle length of the vastus lateralis and rectus femoris muscles, and pennation angle of the rectus femoris muscle. However, the study suffers from several methodological flaws pertaining to the included studies, which would lead to biased outcomes. For example, some studies have used plyometric jump training with increased or reduced body mass (Hirayama et al., 2017; Ullrich et al., 2018; Stien et al., 2020), others used a combination of plyometric jump training with traditional resistance training (Hunter and Marshall, 2002; Kijowksi et al., 2015) and some of the included studies actually did not use plyometric training at all (Helland et al., 2017; Horwath et al., 2019; Kudo et al., 2020; van der Zwaard et al., 2021). Additionally, the authors calculated within-group pre-post effect size (Ramírez-delaCruz et al., 2022). Of note, such an approach has been criticized as it results in biased outcomes (Cuijpers et al., 2017).

Given the inconsistent outcomes as to the effects of plyometric jump training on skeletal muscle hypertrophy from individual studies and the methodological shortcomings in a recent meta-analysis (Ramírez-delaCruz et al., 2022), there is a need to systematically summarize the literature and aggregate data from the available studies to draw more conclusive evidence (Higgins, 2011). Additionally, factors such as age, sex, and training experience as well as different training variables like weekly session frequency, number of jumps per session, and training duration appear to moderate the effects of plyometric jump training on measures of physical fitness (de Villarreal et al., 2009; Sáez-Sáez de Villarreal et al., 2010; Asadi et al., 2016), yet are not well elucidated for skeletal muscle hypertrophy. Therefore, the primary aim of this systematic review with multilevel meta-analysis was to examine the effect of plyometric jump training on skeletal muscle hypertrophy in healthy individuals. The secondary objective was to identify the factors (i.e., age, sex, and training experience) and plyometric jump training variables (i.e., total training period, weekly session frequency, single session duration, and number of jumps per week) that potentially moderate the effect on skeletal muscle hypertrophy to help guide training prescription.

## 2 Materials and Methods

This systematic review was conducted per the Preferred Reporting Items for Systematic Review and Meta-analysis (PRISMA) statements (Page et al., 2021). The current study was pre-registered in the International Prospective Register of Systematic Reviews (PROSPERO) on 13 August 2021 under the registration number “CRD42021265213.”

### 2.1 Search Strategy

A literature search was performed separately and independently by two of the coauthors (AF and CH) in PubMed, SPORTDiscus, Web of Science, and Cochrane Library databases up to September 2021. The search was conducted using a Boolean search strategy with the operators “AND” and “OR” and a combination of the following keywords: (“plyometric” OR “stretch-shortening cycle” OR “stretch shortening cycle*” OR “jump training” OR jump) AND (“hypertrophy” OR “muscle size” OR “muscle mass” OR “muscle fiber” OR “muscle fibre” OR “lean body mass” OR “fat-free mass” OR “cross-sectional area” OR “quadriceps size” OR “circumference”). Keywords were determined through expert opinion, literature review, and controlled vocabulary (e.g., Medical Subject Headings). In addition, corresponding meta-analyses, as well as studies that were eligible for inclusion, were searched for additional publications in so-called “snowball” searches (Greenhalgh and Peacock, 2005). Only peer-reviewed studies written in English were considered for inclusion.

### 2.2 Inclusion and Exclusion Criteria (Study Selection)

We used the PICOS (Population, Intervention, Comparison, Outcome, Study Design) approach to identify eligible studies (Moher et al., 2009). The following inclusion criteria were defined a priori: 1) Population: a cohort of healthy participants with no restriction related to age, sex, or training experience, 2) Intervention: plyometric jump training (i.e., jump exercises soliciting the stretch-shortening cycle), with a minimum duration of 4 weeks (Monti et al., 2020), 3) Comparison: active/passive control group, 4) Outcome: at least one measure of skeletal muscle hypertrophy (e.g., muscle/single fiber cross-sectional area, muscle thickness, lean body mass, muscle circumference), and 5) Study design: (randomized) controlled trials with baseline and follow up measures. We excluded studies involving individuals with pre-existing health problems (e.g., diabetes, hypertension, and asthma), an absence of a passive/active control group, plyometric jump training interventions in combination with additional interventions (e.g., nutrition), and/or lack of baseline and follow-up data.

### 2.3 Data Extraction

The first author (AF) extracted the data from the included studies in a standardised template created with Microsoft Excel. A second author (MA) cross-verified the extracted data. In case of disagreement pertaining data extraction or study eligibility, co-author CH was consulted for clarification.

Of note, we included data for all skeletal muscle hypertrophy measures reported in the studies and for all time points at which they were measured. More specifically, if a study reported multiple skeletal muscle hypertrophy measures, they were all included, and if a study reported measurements during and after the training period, they were all included as well (dependence between effect sizes was handled using a multilevel robust variance estimation approach—see statistical analyses). If data were not reported in a way that was conducive to extraction for our analysis, we contacted the respective authors to request appropriate data [i.e., mean ± standard deviation (SD), raw data]. When multiple studies were published using the same data set (Skurvydas and Brazaitis, 2010; Skurvydas et al., 2010), only one study was considered (Skurvydas and Brazaitis, 2010). In cases where the authors did not respond to our request for raw data, we used WebPlotDigitizer (v4.3, Ankit Rohatgi; https://apps.automeris.io/wpd/) to extract relevant data in studies that only reported graphical data (Drevon et al., 2017).

From all included studies, we extracted 1) author and year of publication; 2) mean age of participants; 3) percentage of females in the sample; 4) training experience [i.e., athlete vs. non-athlete (see footnote^1^)], 5) muscle group investigated [i.e., knee extensors (e.g., vastus medialis and rectus femoris) vs., plantar flexors (e.g., gastrocnemius)], 6) assessment method (i.e., ultrasound and prediction equation), 7) total training period (weeks), 8) weekly session frequency, 9) single session duration, 10) total number of jumps per week, and 11) type of jumping. The characteristics of the included studies are displayed in Tables 1, 2.

**TABLE 1:** Characteristics of the population and assessment methods of included studies.

Author	Population	Assessment method					
	*N*	Percent female (%)	Mean age (years)	Training experience	Outcome	Tool	Muscle group
Allison et al. (2018)	int [20]/con [15]	0	72.5	Non-athletes	Muscle thickness	B-mode ultrasound	plantar flexor [gastrocnemius]
Chelly et al. (2010)	int [12]/con [11]	0	19	Athletes	CSA	Prediction equation	Unspecified
					Muscle volume	Prediction equation	Unspecified
Chelly et al. (2014)	int [12]/con [11]	0	17.4	Athletes	Muscle volume	Prediction equation	Unspecified
Chelly et al. (2015)	int [14]/con [13]	0	11.9	Athletes	CSA	Prediction equation	Unspecified
					Muscle volume	Prediction equation	Unspecified
Cherni et al. (2020)	int [15]/con [12]	100	21	Athletes	CSA	Prediction equation	Unspecified
					Muscle volume	Prediction equation	Unspecified
Correa et al. (2012)	int [14]/con [17]	0	67	Non-athletes	Muscle thickness	B-mode ultrasound	Knee extensor [vastus lateralis]
Earp et al. (2015)	int [9]/con [9]	0	26.5	Non-athletes	CSA	Ultrasound	Knee extensor [vastus medialis]
Fathi et al. (2019)	int [20]/con [20]	0	14.6	Athletes	Muscle volume	Prediction equation	Knee extensor [rectus femoris]
Fouré et al. (2011)	int [9]/con [10]	0	18.8	Non-athletes	CSA	Ultrasound	Knee extensor [quadriceps femoris]
Fouré et al. (2012)	int [9]/con [10]	0	20.9	Non-athletes	CSA	Ultrasound	Knee extensor [vastus lateralis]
Herrero et al. (2006)	int [9]/con [10]	0	20.1	Non-athletes	CSA	Prediction equation	Knee extensor [vastus intermedialis]
Kyröläinen et al. (2005)	int [13]/con [10]	0	24.4	Non-athletes	Fiber size I	Biopsy	Knee extensor [vastus medialis]
					Fiber size IIA	Biopsy	Knee extensor [rectus femoris]
					Fiber size IIAX	Biopsy	Unspecified
					Fiber size IIX	Biopsy	Plantar flexor [triceps surae]
Marković et al. (2005)	int [50]/con [51]	0	21	Non-athletes	Calf girth	Tape	Plantar flexor [gastrocnemius]
					Thigh girth	Tape	Unspecified
McKinlay et al. (2018)	int [13]/con [14]	0	12	Non-athletes	Muscle thickness	B-mode ultrasound	Plantar flexor [gastrocnemius]
Skurvydas and Brazaitis, (2010)	int [13]/con [10]	0	10.3	Non-athletes	Muscle thickness	Ultrasound	Plantar flexor [gastrocnemius]
		56.52	10.3	Non-athletes	Muscle thickness	Ultrasound	Knee extensor [quadriceps]

int, intervention; con, control condition; PJT, plyometric jump training; CSA, cross-sectional area.

**TABLE 2:** Characteristics of the plyometric jump training interventions of included studies.

Author	Intervention characteristics						
	Intervention	Control	Total training period (weeks)	Session duration (mins)	Weekly Session frequency (mean)	Jumps per week (mean)	Total number of jumps
Allison et al. (2018)	PJT [unilateral; OLH]	Regular training	26	5	7	350	9,100
Chelly et al. (2010)	PJT [bilateral; DJ/HJ]	Regular training	8	30	2	107.5	860
Chelly et al. (2014)	PJT [bilateral; DJ/HJ]	Regular training	8	30	2	107.5	860
Chelly et al. (2015)	PJT [bilateral; DJ/HJ]	Regular training	10	20	2	120	1,200
Cherni et al. (2020)	PJT [bilateral; DJ/HJ]	Regular training	8	17.25	2	199.5	1,596
Correa et al. (2012)	PJT [bilateral; LBJ]	Regular training	6	NA	NA	NA	NA
Earp et al. (2015)	PJT [bilateral; SJ]	Regular training	8	NA	3	99	792
Fathi et al. (2019)	PJT [bilateral; DJ/HJ]	Regular training	16	NA	2	74	1,184
Fouré et al. (2011)	PJT [bilateral; CMJ/DJ/HJ/SJ]	Regular training	14	60	2.43	486	6,804
Fouré et al. (2012)	PJT [bilateral; CMJ/DJ/HJ/SJ]	Regular training	14	60	2.43	486	6,804
Herrero et al. (2006)	PJT [bilateral; DJ/HoJ]	Regular training	4	25	2	195	780
Kyröläinen et al. (2005)	PJT [bilateral; DJ/HJ/OLH/SJ/TLH]	Regular training	15	NA	2	NA	NA
Marković et al. (2005)	PJT [bilateral; DJ/HJ]	Regular training	10	60	3	180	1800
McKinlay et al. (2018)	PJT [bilateral; CMJ/KCJ/DJ/HoJ/OLH/TLH/other]	Regular training	8	30	3	519.75	4,158
Skurvydas and Brazaitis, (2010)	PJT [bilateral; CMJ]	Regular training	8	NA	2	60	480

PJT, plyometric jump training; OLH, one leg hopping; HJ, hurdle jump; DJ, drop jump; LBJ, lateral box jump; SJ, squat jump; CMJ, countermovement jump; HoJ, horizontal jump; KTJ, knee to chest jump.

### 2.4 The Methodological Quality of the Included Studies

The Physiotherapy Evidence Database (PEDro) scale was used to evaluate the methodological quality of the included studies. The validity and reliability of the PEDro scale have been established in previous studies (Maher et al., 2003; de Morton, 2009). Additionally, its agreement with other assessment tools such as the Cochrane risk of bias tool has been established (Moseley et al., 2019). As blinding of participants and investigators is not feasible in exercise interventions and blinding of assessors is rarely implemented, items 5–7 were removed from the scale consistent with previous systematic reviews (Grgic et al., 2017; Schoenfeld et al., 2017). Hence the methodological quality of the included studies was rated on a scale from 0 to 7. In accordance with previous systematic reviews (Kümmel et al., 2016; Schoenfeld et al., 2017), the quality of the included studies was categorized as “poor” = 0–3, “moderate” = 4, “good” = 5, and “excellent” = 6–7 (Table 3). Additionally, to visually estimate publication bias, a contour-enhanced funnel plot was used (Harrer et al., 2019).

**TABLE 3:** Modified Physiotherapy Evidence Database (PEDro) scores of the reviewed studies.

Author	1*	2	3	4	8	9	10	11	Score
Allison et al. (2018)	+	−	−	+	−	+	+	+	4
Chelly et al. (2010)	−	+	−	+	+	+	+	+	6
Chelly et al. (2014)	−	+	−	+	+	+	+	+	6
Chelly et al. (2015)	+	+	−	+	+	+	+	+	6
Cherni et al. (2020)	+	−	−	+	+	+	+	+	5
Correa et al. (2012)	+	+	−	+	+	+	+	+	6
Earp et al. (2015)	+	+	−	+	+	+	+	+	6
Fathi et al. (2019)	+	+	−	+	+	+	+	+	6
Fouré et al. (2011)	−	+	−	+	+	+	+	+	6
Fouré et al. (2012)	−	−	−	+	+	+	+	+	5
Herrero et al. (2006)	+	+	−	+	+	+	+	+	6
Kyröläinen et al. (2005)	−	+	−	+	+	+	+	+	6
Marković et al. (2005)	+	+	−	+	+	+	+	+	6
McKinlay et al. (2018)	+	−	−	+	+	+	+	+	5
Skurvydas and Brazaitis, (2010)	+	−	−	−	+	+	+	+	4
Median									6

*The first criterion was excluded for the calculation of the PEDro score; + indicates a “yes” score; − indicates a “no” score.

Because blinding of participants and investigators is impossible in supervised exercise interventions and blinding of assessors is rarely implemented, items 5–7 were removed from the scale.

### 2.5 Synthesis and Analyses

The meta-analysis was performed using the “metafor” (Viechtbauer, 2010) and “tidyverse” (Wickham et al., 2019) packages in R (v 4.0.2; R Core Team, https://www.r-project.org/). All analyses are available in the supplementary documentation (https://osf.io/bf478/). The standardised mean difference (SMD) was calculated by subtracting the standardised mean change of the intervention group minus the standardised mean change of the control group. The respective variance was calculated by pooling the pre-test standard deviations of both groups, an approach deemed appropriate to provide a comparatively unbiased estimate of the population effect size (Morris, 2008). The magnitude of standardised effect sizes was interpreted in accordance with Cohen’s thresholds (Cohen, 1988): trivial (< 0.2), small (0.2 to 0.5), moderate (0.5 to 0.8), and large (≥ 0.8).

Due to the nested structure of the calculated effect sizes (i.e., effects nested within groups nested within studies), multilevel mixed-effects meta-analyses with study and intra-study groups as random effects were calculated to examine the effect of plyometric jump training on skeletal muscle hypertrophy. Further, cluster robust point estimates using 95% compatibility (confidence) intervals (CIs) were calculated (Hedges et al., 2010) and weighted by inverse sampling variance to account for the within- and between-study variance (tau-squared). Restricted maximal likelihood estimation was applied in all models. A main model was created containing all effect sizes. Additional exploratory subgroup comparisons and meta-regression analyses of moderator variables were performed, including mean age, proportion of females per group, training experience, and muscle group studied, as well as training characteristics such as total training period, training frequency, session duration, and number of jumps per week. For training experience and muscle group examined, multilevel models with subgroups were calculated and robust estimates were produced. Meta-regressions were calculated for mean age, proportion of females per study, and the aforementioned training characteristics.

To avoid dichotomizing the existence of an effect in our models, we reported absolute *p*-values but did not employ traditional null hypothesis significance testing (Amrhein et al., 2019a; Amrhein et al., 2019b; McShane et al., 2019). We also focused on the point estimate in the interpretation with the greatest emphasis on the effects from the lower to the upper limit of the interval estimates (Nakagawa and Cuthill, 2007; Lee, 2016; Van Calster et al., 2018). The risk of small study bias was visualised through contour-enhanced funnel plots. Further, Q and I^2^ statistics were produced and reported (Higgins et al., 2003). A significant Q statistic is usually taken as an indicator that the effects are unlikely to come from a common population. I^2^ values indicate the degree of heterogeneity of effects as follows: 0%–40% indicates no heterogeneity, 30%–60% moderate heterogeneity, 50%–90% substantial heterogeneity, and 75%–100% considerable heterogeneity (Higgins et al., 2019). For within-participant effects, pre-post correlations for measures have rarely been reported; therefore, we adopted a range of values for correlation coefficients (*r* = 0.5, 0.7, and 0.9) and examined the sensitivity of the results to each of these values. Since the overall results were relatively insensitive to this range, we reported the results for *r* = 0.7 here and included the results for the other assumed correlation coefficients in the Supplementary Material is available on the following link: (https://osf.io/bf478/).

## 3 Results

### 3.1 Study Characteristics

**FIGURE 1:**
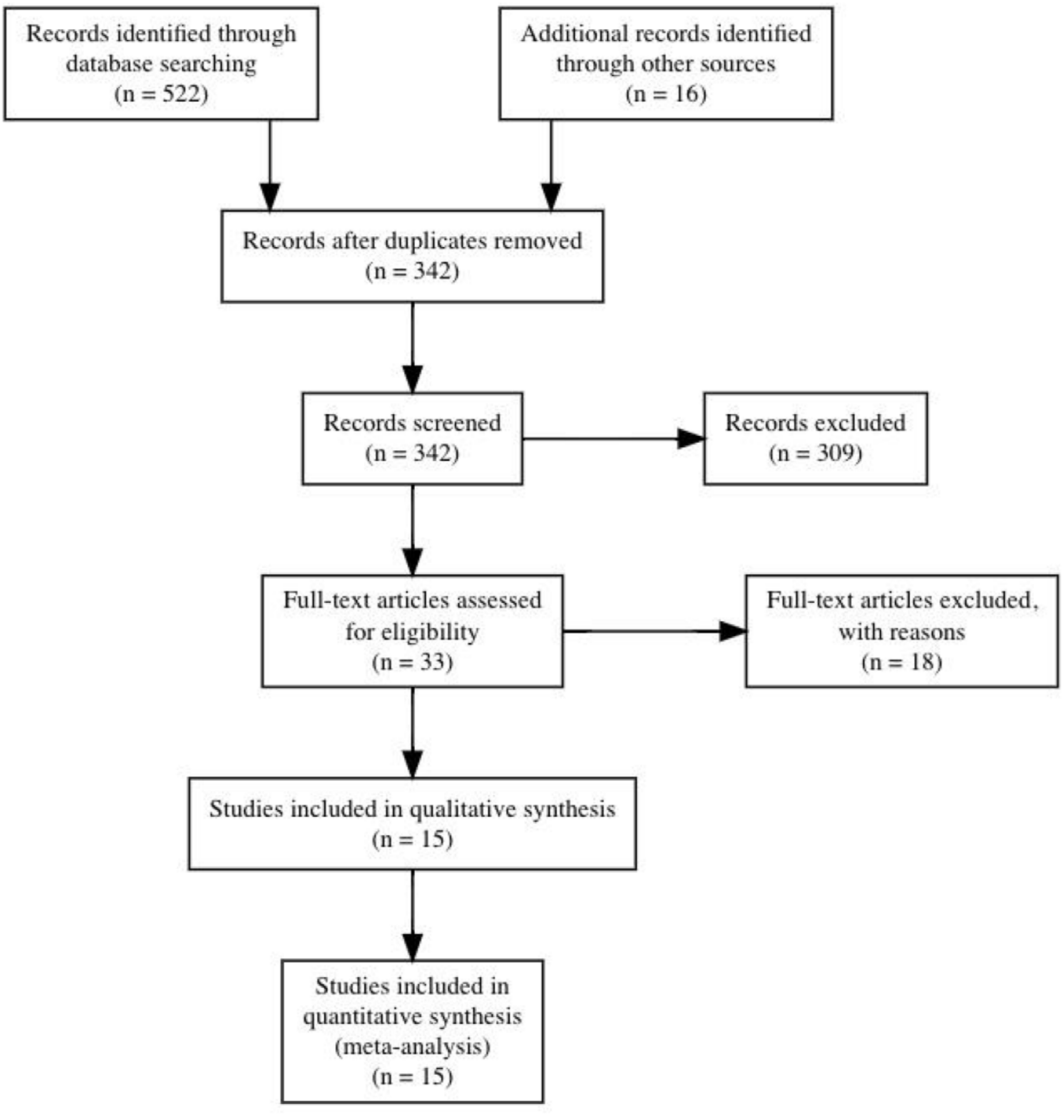
Flow chart illustrating the different stages of search and study selection.

After initial searches and screening, nine studies were identified that met inclusion criteria. Supplementary search approaches identified six additional eligible studies. Thus, a total of 15 studies were ultimately included for analysis. All studies included active control groups. Details of the search and inclusion process are shown in the flow chart (Figure 1; https://osf.io/3u567/). The total number of participants across the included studies is 478 (range = 18–101; median = 23), comprising 245 INT (range = 9–50, median = 13) and 233 CON (range = 5–51, median = 11). The majority of the studies used different types of jumps in their training programmes (e.g., bilateral/unilateral vertical and horizontal jumps) (Kyröläinen et al., 2005; Marković et al., 2005; Herrero et al., 2006; Chelly et al., 2010; Fouré et al., 2011; Fouré et al., 2012; Chelly et al., 2014; Chelly et al., 2015; McKinlay et al., 2018; Fathi et al., 2019; Cherni et al., 2020). Four studies used one single type of jump such as bilateral countermovemt jump or lateral box jump (Skurvydas and Brazaitis, 2010; Correa et al., 2012; Earp et al., 2015; Allison et al., 2018). The mean age across studies ranged from 10.3 to 72.5 years with a median of 20.1 years. Two studies examined the effects of plyometric jump training on hypertrophy in female participants (Correa et al., 2012; Cherni et al., 2020), while one study included a mixed intervention group but used a male-only control group (Skurvydas and Brazaitis, 2010). The remaining twelve studies included male participants. Two studies included older adults (Correa et al., 2012; Allison et al., 2018). As to training experience, five studies recruited athletes (Chelly et al., 2010; Chelly et al., 2014; Chelly et al., 2015; Fathi et al., 2019; Cherni et al., 2020), while ten studies investigated the effects of plyometric jump training on skeletal muscle hypertrophy in non-athletes (Kyröläinen et al., 2005; Marković et al., 2005; Herrero et al., 2006; Skurvydas and Brazaitis, 2010; Fouré et al., 2011; Fouré et al., 2012; Correa et al., 2012; Earp et al., 2015; Allison et al., 2018; McKinlay et al., 2018). Regarding the muscle group investigated, four studies examined hypertrophy in the knee extensors (Skurvydas and Brazaitis, 2010; Correa et al., 2012; Earp et al., 2015; McKinlay et al., 2018) and four in the plantar flexor (Kyröläinen et al., 2005; Fouré et al., 2011; Fouré et al., 2012; Allison et al., 2018). In the seven remaining studies, the muscle group investigated was not specified (e.g., assessment of thigh muscle volume or thigh/calf girth) (Marković et al., 2005; Herrero et al., 2006; Chelly et al., 2010; Chelly et al., 2014; Chelly et al., 2015; Fathi et al., 2019; Cherni et al., 2020). Seven studies used ultrasound imaging technique (Skurvydas and Brazaitis, 2010; Fouré et al., 2011; Fouré et al., 2012; Correa et al., 2012; Earp et al., 2015; Allison et al., 2018; McKinlay et al., 2018), while six studies used a prediction equation to assess muscle hypertrophy (Herrero et al., 2006; Chelly et al., 2010; Chelly et al., 2014; Chelly et al., 2015; Fathi et al., 2019; Cherni et al., 2020). One study assessed muscle hypertrophy using muscle biopsy (Kyröläinen et al., 2005) and one study used tape (Marković et al., 2005). The median duration of plyometric jump training was 8 weeks (range from 4–26) and the median weekly session frequency was two (range from 2–7). The median session duration was 30 min and ranged from 5 to 60 min. However, session duration was not reported in two studies (Kyröläinen et al., 2005; Correa et al., 2012). The median of the number of jumps performed per week was 230 (range = 60–520). Full details of all included studies can be seen in Tables 1, 2. Regarding the methodological quality of the included studies, the PEDro scores ranged from 4 to 6 with a median score of 6 (Table 3).

### 3.2 Main Model—All Effects

**FIGURE 2:**
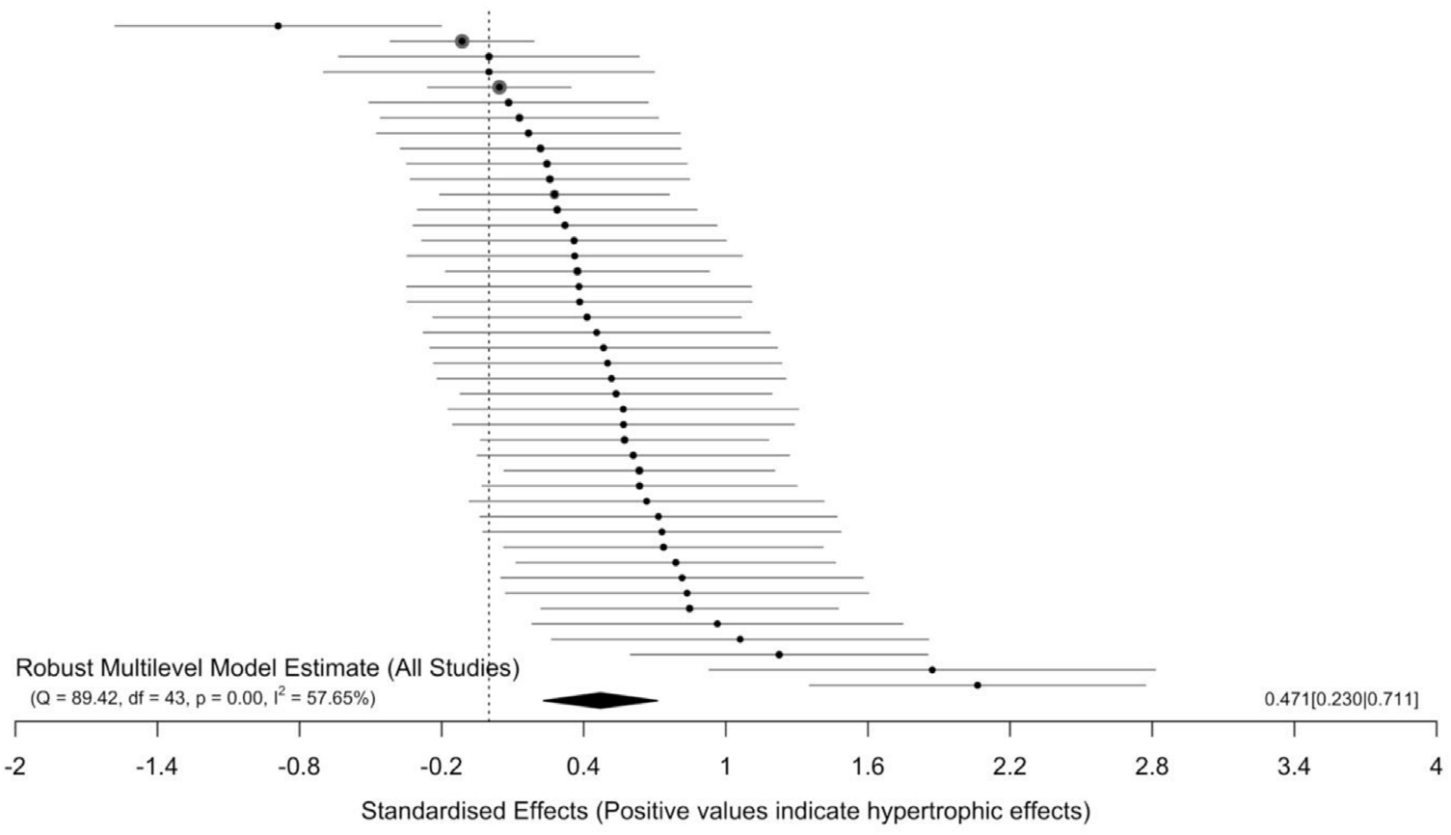
Ordered caterpillar plot of all effects.

**FIGURE 3:**
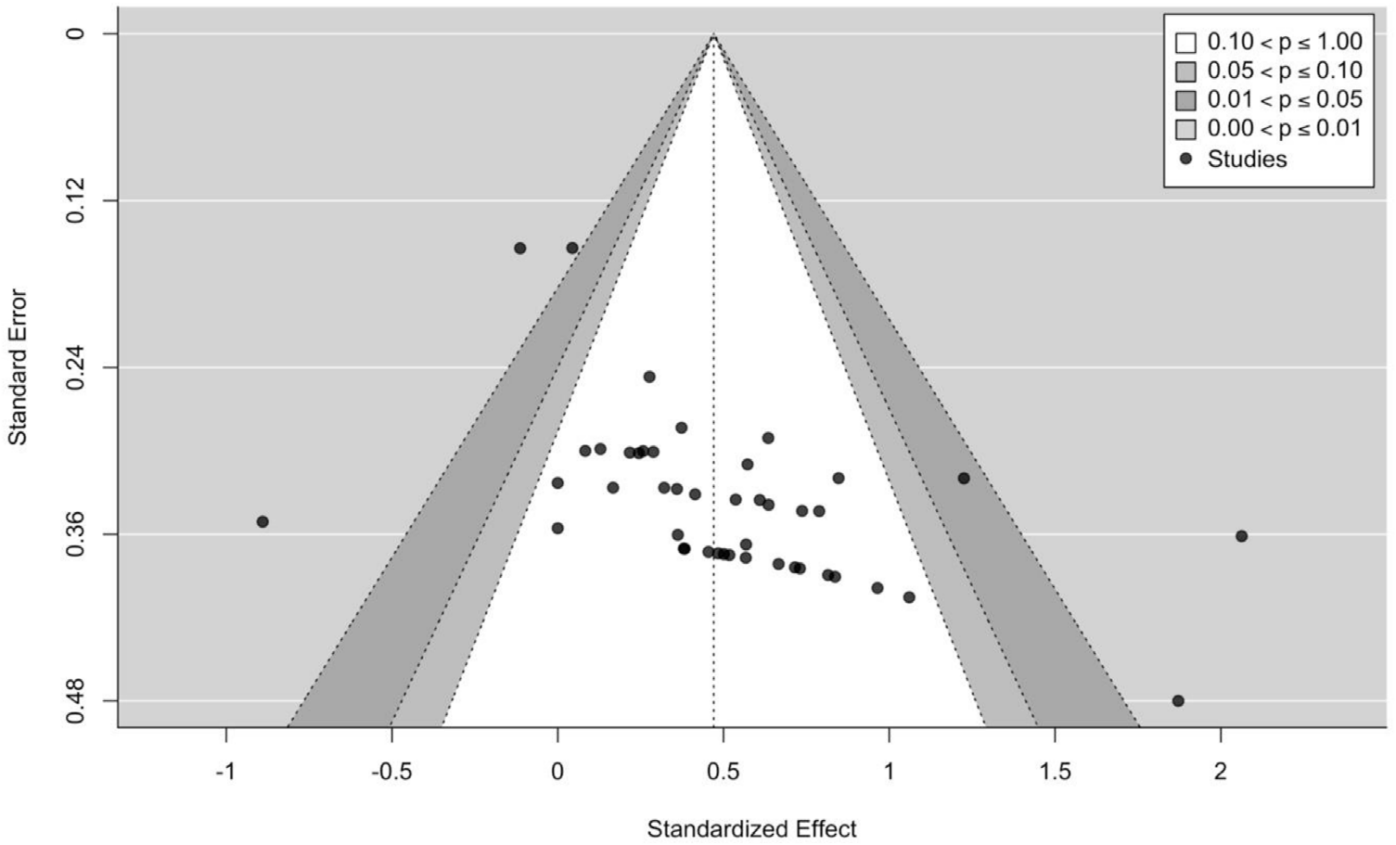
Contour enhanced funnel plot for all effects.

The main model (44 effect sizes across 15 clusters median = 2, range = 1–15 effects per cluster) yielded small to moderate effects with a small point estimate [SMD = 0.47 (95% CIs = 0.23–0.71); *p* < 0.001] and moderate to substantial heterogeneity (I^2^ = 57.53%). All effect sizes and interval estimates are presented in an ordered caterpillar plot (Figure 2; https://osf.io/csnrq/). The visual inspection of the funnel plot indicated a seemingly symmetrical distribution pattern of the effects that might be reflective of an apparently absence of publication bias (Figure 3; https://osf.io/gv7xq/).

### 3.3 Subgroup and Meta-Regression Analyses

#### 3.3.1 Muscle Group

**FIGURE 4:**
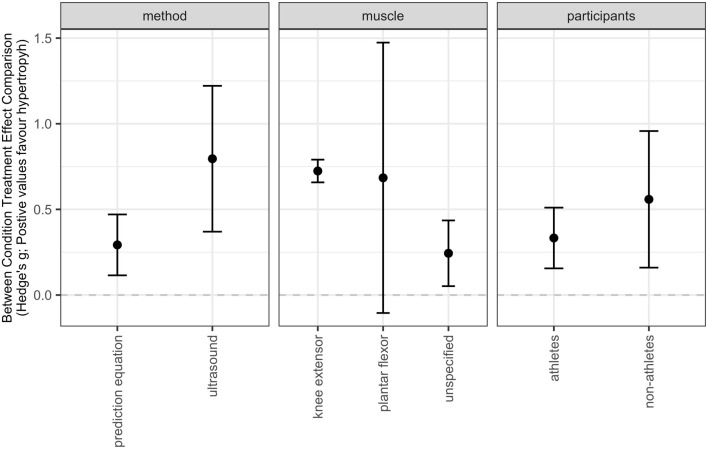
Point-range plots of categorical subgroups on skeletal muscle hypertrophy.

Subgroup models showed moderate effects for the knee extensors with a moderate point estimate [SMD = 0.72 (95% CIs = 0.66–0.78); *p* < 0.001] and an equivocal effect for the plantar flexors with a moderate point estimate [SMD = 0.64 (95% CIs = −0.25 to 1.55); *p* = 0.142]. Additionally, trivial to moderate effects with a small point estimate were observed for unspecified muscle groups [SMD = 0.23 (95% CIs = 0.04–0.43); *p* = 0.024]. The difference between sub-groups was notable (*p* = 0.001). The level of heterogeneity was moderate (I^2^ = 48.25%). This subgroup analysis is presented in a point-range plot in Figure 4 (https://osf.io/wn2xv/).

#### 3.3.2 Training Experience

Subgroup models indicated trivial to moderate effects with a small point estimate for athletes [SMD = 0.33 (95% CIs = 0.16–0.51); *p* = 0.001] and trivial to large effects with a moderate point estimate for non-athletes [SMD = 0.55 (95% CIs = 0.18–0.93); *p* = 0.007] with no differences between subgroups (*p* = 0.270). The level of heterogeneity was substantial (I^2^ = 58.84%). The subgroup analysis is presented in a point-range plot in Figure 4 (https://osf.io/wn2xv/).

#### 3.3.3 Assessment Method

Subgroup models revealed trivial to small effects with a small point estimate for prediction equations [SMD = 0.29 (95% CIs = 0.16–0.42); *p* < 0.001] and moderate to large effects with a moderate point estimate for ultrasound imaging [SMD = 0.74 (95% CIs = 0.59–0.89); *p* < 0.001] with a clear difference between subgroups (*p* < 0.001). Analysis further revealed no heterogeneity (I^2^ = 0%). The subgroup analysis is presented in a point-range plot in Figure 4 (https://osf.io/wn2xv/).

#### 3.3.4 Mean Age of Participants

**FIGURE 5:**
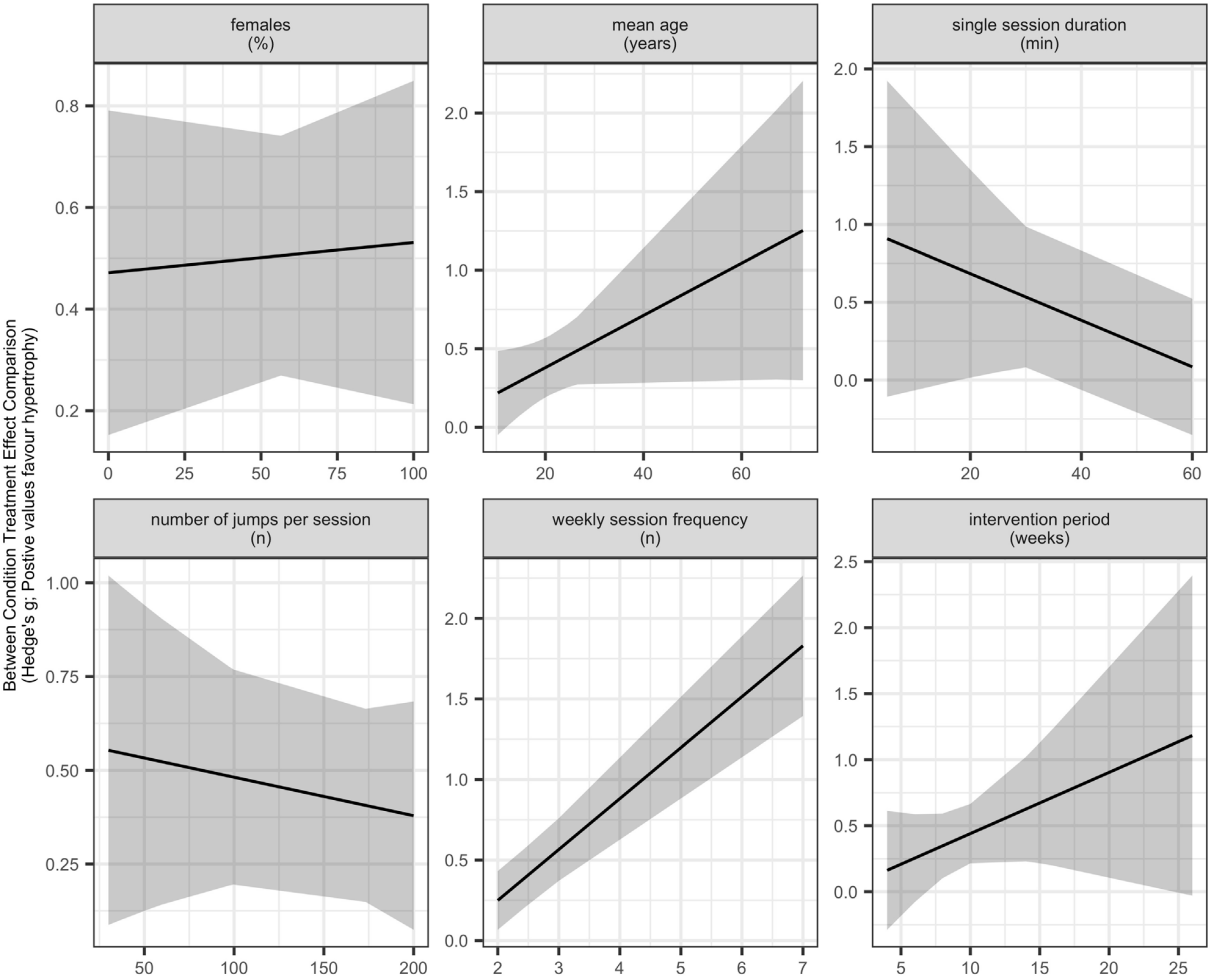
Meta-analytic plots of continuous subgroups on skeletal muscle hypertrophy.

Meta-regression analyses did not indicate clear evidence that age moderates skeletal muscle hypertrophy adaptations in response to plyometric jump training [β = 0.0149 (95% CIs = −0.0033 to 0.0332); *p* = 0.101]. The level of heterogeneity was moderate (I^2^ = 46.90%). The regression analysis is depicted in a meta-analytic plot in Figure 5 (https://osf.io/g8aet/).

#### 3.3.5 Percentage of Females in the Sample

Meta-regression analyses did not detect clear evidence that the percentage of females in the sample moderates the effects of plyometric jump training on skeletal muscle hypertrophy [β = 0.0006 (95% CIs = −0.0036 to 0.0049); *p* = 0.751]. The level of heterogeneity was substantial (I^2^ = 60.43%). The subgroup analysis is presented in a meta-analytic plot in Figure 5 (https://osf.io/g8aet/).

#### 3.3.6 Total Training Period

Meta-regression analyses did not indicate clear evidence that total training period moderates the effect of plyometric jump training on skeletal muscle hypertrophy [β = 0.0433 (95% CIs = −0.0347 to 0.1215); *p* = 0.253). The level of heterogeneity was moderate to substantial (I^2^ = 56.78%). The subgroup analysis is illustrated in a meta-analytic plot in Figure 5 (https://osf.io/g8aet/).

#### 3.3.7 Weekly Session Frequency

Meta-regression analyses showed that the weekly session frequency moderates the effect of plyometric jump training on skeletal muscle hypertrophy, with more sessions per week inducing larger gains in hypertrophy [β = 0.3233 (95% Cis = 0.2040–0.4425); *p* < 0.001]. The analysis further revealed no to moderate heterogeneity (I^2^ = 38.63%). The subgroup analysis is presented in a meta-analytic plot in Figure 5 (https://osf.io/g8aet/).

#### 3.3.8 Single Session Duration

Meta-regression analyses revealed no clear evidence that single session duration moderates the effects of plyometric jump training on skeletal muscle hypertrophy [β = −0.0133 (95% CIs = −0.0387 to 0.0120); *p* = 0.261). The analysis further revealed substantial heterogeneity (I^2^ = 70.36%). The subgroup analysis is depicted in a meta-analytic plot in Figure 5 (https://osf.io/g8aet/).

#### 3.3.9 Number of Jumps Per Week

Meta-regression analyses revealed no clear evidence that the number of jumps per week moderates the effects of plyometric jump training on skeletal muscle hypertrophy [β = 0.0009 (95% CIs = −0.0006 to 0.0025) *p* = 0.214]. The analysis showed substantial heterogeneity (I^2^ = 60.57%). The subgroup analysis is presented in a meta-analytic plot in Figure 5 (https://osf.io/g8aet/).

## 4 Discussion

This systematic review with multilevel meta-analysis aimed to 1) examine the effects of plyometric jump training on skeletal muscle hypertrophy in healthy individuals across different age ranges and 2) identify potentially important plyometric jump training variables relevant for promoting hypertrophic adaptations to help guide training prescription. The main findings indicated that plyometric jump training elicits small to moderate effects on skeletal muscle hypertrophy, regardless of sex, age, or training experience. Additionally, subgroup analyses showed relatively larger effects in non-athletes compared with athletes, and moderate effects for the knee extensors with an equivocal effect for the plantar flexors. Moreover, we found no clear evidence that age or sex moderated the effects of plyometric jump training on skeletal muscle hypertrophy. Furthermore, meta-regression analyses suggested that the effects on skeletal muscle hypertrophy were moderated by the weekly session frequency, with more frequent weekly plyometric jump training sessions resulting in larger hypertrophic adaptations. We found no clear evidence that total training period, single session duration, or the number of jumps per week moderate the effects of plyometric jump training on skeletal muscle hypertrophy.

### 4.1 Main Effect

The main findings of the present meta-analysis indicated small to moderate effects of plyometric jump training on skeletal muscle hypertrophy [SMD = 0.47 (95% CIs = 0.23–0.71)], regardless of sex, age, and training experience. The current outcomes corroborate the results of a recently published systematic review with meta-analysis where authors reported a moderate effect of plyometric jump training on muscle thickness (SMD = 0.59) and fascicle length (SMD = 0.51) in healthy adults (Ramírez-delaCruz et al., 2022). However, our results are relatively more conservative, which we attribute to our more stringent inclusion criteria for plyometric jump training as well as the comparison of the plyometric jump training to an active/passive control group. In sum, the findings of the current study as well as those of recent ones (Grgic et al., 2020; Ramírez-delaCruz et al., 2022) question the common belief, indicating that plyometric jump training can indeed increase not only the motor drive to the active muscles but also skeletal muscle hypertrophy.

The previously claimed limited potential for plyometric jump training-related hypertrophic adaptations has been attributed to the relatively short time under tension during the jumps and, therefore, a reduced mechanical stimulus for muscle protein synthesis (Schoenfeld, 2010; Wackerhage et al., 2019). In addition, some researchers have proposed that the inability to continually provide an overload stimulus during plyometric jump training is another potential limitation from a hypertrophy standpoint (Suchomel et al., 2018). In this context, while extra loads additional to body mass may be used with plyometric jump exercises (e.g., weighted vests) (Negra et al., 2020), caution must be taken given that heavier loads may result in greater impact forces and delay the transition time between eccentric and concentric muscle actions, which could harm the overall training stimulus (Suchomel et al., 2018). However, our findings seem to refute these claims. Indeed, the small to moderate effects of plyometric jump training on skeletal muscle hypertrophy observed in this study indicate that the high contraction velocity seems to contribute to skeletal muscle hypertrophy.

Earlier studies showed that high-velocity lengthening actions (i.e., rapid eccentric phase during movements under the stretch-shortening cycle) tend to hypertrophy type II compared with type I muscle fibers (Potteiger et al., 1999; Shepstone et al., 2005; Malisoux et al., 2006b). For example, Shepstone et al. (2005) studied the effects of two modes of resistance training programmes, fast vs. slow isokinetic lengthening action of the elbow flexors, on muscle fiber hypertrophy in healthy untrained individuals aged 24 years. The results of the study demonstrated greater hypertrophy in type IIa muscle fibers following fast (+13%) compared with slow muscle lengthening (+3%). Authors further demonstrated greater (+185%) Z-line disruption following fast compared with slow muscle lengthening. It should be noted that Z-line disruption is considered a prominent marker of muscle protein remodeling (Yu and Thornell, 2002; Yu et al., 2004). Additionally, there is direct evidence based on muscle biopsy that a single bout of plyometric exercise induces preferential damage (e.g., loss in dystrophin staining, Z-line disruption) to type II muscle fibers (Macaluso et al., 2012). Of note, exercise-induced damage to muscle tissues is discussed as a potential mechanism for skeletal muscle hypertrophy, perhaps mediated by stimulating satellite cell activity (Vierck et al., 2000; Schoenfeld, 2010; Schoenfeld, 2012). In fact, satellite cells represent the resident stem cells of skeletal muscle (Scharner and Zammit, 2011) and lead to increased muscle regeneration (Barton-Davis et al., 1999). Existing evidence indicated larger satellite cell activation and proliferation after exercise that induced muscle damage (Crameri et al., 2007).

Exercise training needs to cause positive net protein balance to induce skeletal muscle hypertrophy. To our knowledge, muscle protein synthetic responses to plyometric jump training in humans have never been examined in the literature (Grgic et al., 2020). Relevant outcomes from an animal study indicated that rats exposed to plyometric jump training showed a positive net protein balance compared with a control condition (Watt et al., 1982). Lim et al. (2017) examined the effects of one bout of single-mode traditional resistance training vs. one bout of combined traditional resistance training and plyometric jump training on satellite cell activity and anabolic signaling in elite male weightlifters. Their results revealed an increase in satellite cell activation and myofibrillar protein synthesis following both exercise modes. However, the same authors reported that single-mode traditional resistance training resulted in higher satellite cell activity with a tendency for higher expression of mTOR (mammalian target of rapamycin) and p70S6K (ribosomal protein S6 kinase) compared with combined traditional resistance training and plyometric jump training (Lim et al., 2017). Despite these intriguing results, this study does not provide insights into the effects of single-mode plyometric jump training on the anabolic signaling pathway. Therefore, researchers should seek to fill this gap in the literature. Overall, contrary to the previous speculation, plyometric jump training appears to contribute to skeletal muscle hypertrophy, regardless of sex, age, and training experience. These findings have both important scientific and practical implications.

### 4.2 Moderating Variables

Our findings indicate moderate effects of plyometric jump training on knee extensor hypertrophy [SMD = 0.72 (95% CIs = 0.66–0.78)]. However, the heterogeneity of the outcomes across studies yielded an equivocal effect of plyometric jump training on plantar flexor hypertrophy (SMD = 0.64; 95% CIs = −0.25 to 1.55). It has been shown that jumping exercises principally solicit activation of the knee extensors (i.e., quadriceps) and plantar flexors (e.g., gastrocnemius) but not hamstrings (Ebben et al., 2008). As such, we would expect larger hypertrophic adaptations in these muscles. Monti et al. (2020) investigated the effects of 6 weeks of plyometric jump training on knee extensor muscle mass in healthy males aged 25 years. They demonstrated increased knee extensor power (+19.7%), which was accompanied by increases in quadriceps femoris (+5.8%) and vastus lateralis (+9.6%) volume as well as mean CSA of the quadriceps femoris (+5.8%) after plyometric jump training. The same authors reported significant positive correlations between mean CSA and volume of quadriceps femoris and muscle power (*R* ^2^ = 0.46 and 0.44, respectively). Additionally, McKinlay et al. (2018) examined the effects of 8 weeks of plyometric jump training on knee extensor hypertrophy and found an 8.1% post-study increase in vastus lateralis muscle thickness in adolescent soccer players aged 11–13 years. Furthermore, Váczi et al. (2014) indicated a 20.5% increase in quadriceps CSA measured *via* MRI in older adults following 10 weeks of plyometric jump training. In sum, plyometric jump training appears to be an effective means to improve knee extensor hypertrophy. Conversely, our findings do not support consistent hypertrophic effects in the plantar flexors. This could be due to the different mechanical properties between the patellar tendon and Achilles tendon, resulting in different hypertrophic effects on the quadriceps (knee extensor) and gastrocnemius (plantar flexor). In fact, the patellar tendon has been shown to be stiffer and, therefore, mechanically better suited to effectively transmit muscle force compared with the Achilles tendon (Wiesinger et al., 2016). Nevertheless, given the relative paucity of research, future studies should be further redirected towards the assessment of plyometric jump training on plantar flexor hypertrophy to achieve more conclusive inferences.

In regard to training experience, results showed a relatively larger effects for non-athletes [0.55 (95% CIs = 0.18–0.93)] compared with athletes [0.33 (95% CIs = 0.16–0.51)], with a substantial degree of heterogeneity observed across studies. It is well-established that previous training history moderates adaptations to further training interventions (Faigenbaum, 2000; Rhea et al., 2003; Harries et al., 2015; Figueiredo et al., 2018; Suchomel et al., 2018). Indeed, a larger magnitude of adaptation to training would be expected in individuals with less, compared with more, training experience (Faigenbaum, 2000; Suchomel et al., 2018). In this context, earlier studies (Rhea et al., 2003; Figueiredo et al., 2018) demonstrated that adaptations to traditional resistance training are moderated by the magnitude of adaptation that has already been achieved by the individual, implying that the so-called “ceiling effect” attenuates continued adaptations. To the authors’ knowledge, none of the available studies have contrasted the effects of plyometric jump training on skeletal muscle hypertrophy of athletes vs. non-athletes, highlighting a void in the current literature. Nevertheless, results from separate studies indicate large effects of plyometric jump training on skeletal muscle hypertrophy (11.5%–18.8% increase of quadriceps femoris CSA) in non-athletes (Earp et al., 2015) but only a relatively small effect in athletes (9.9% increase in thigh CSA) (Cherni et al., 2020). This is in agreement with the findings of the present study, given that trivial to large effects were observed for non-athletes and trivial to moderate effects were noted for athletes. This suggests that to achieve comparable or larger gains, individuals with greater training experience may need to increase their training volume/intensity to a level that exceeds those who are less experienced and/or fit (Suchomel et al., 2018; Chaabene et al., 2020). In summary, plyometric jump training appears to be more effective to improve skeletal muscle hypertrophy in non-athletes compared with athletes. Future studies should seek to explore the specific mechanisms that facilitate larger gains in non-athletes compared with athletes.

For the assessment methods of skeletal muscle hypertrophy, results show larger effects for ultrasound imaging [SMD = 0.74 (95% CIs = 0.59–0.89)] compared with prediction equation [SMD = 0.29 (95% CIs = 0.16–0.42)] with a clear difference between subgroups. Ultrasound is an easy, non-invasive, and rapid tool to assess muscle thickness, which in turn informs about skeletal muscle hypertrophy (Haun et al., 2019). Muscle thickness evaluated using ultrasound is highly reliable in a range of muscles (Thoirs and English, 2009). However, the prediction equation represents a valid tool that affords a crude estimate of skeletal muscle hypertrophy (Chelly et al., 2006). The major drawback of the prediction equation though is that it does not allow for the differentiation between muscle tissue, fat tissue, and bone (Chelly et al., 2006; Grgic et al., 2019). For the reasons above, it is advisable to favor using ultrasound over the prediction equation to provide more accurate insights about skeletal muscle hypertrophy. Nevertheless, the affordability of the prediction equation could make it further useful, when equipment such as ultrasound is not available. Furthermore, the hypertrophic improvement following plyometric jump training seems to be stable across other assessment methods such as biopsies or MRI. For example, Malisoux et al. (2006a) studied the effects of plyometric jump training on single muscle fiber diameters of the vastus lateralis measured using biopsies in healthy active males aged 23 years. Participants demonstrated a significant increase in type I (+11%), type IIa (+10%), and type IIa/IIx (+15%) fibers following training. The same authors reported that fiber force increased for all fiber types, in part due to increased fiber diameter (Malisoux et al., 2006a). Furthermore, Vissing et al. (2008) compared the effects of plyometric jump training vs. traditional resistance training on skeletal muscle hypertrophy *via* MRI in untrained males aged 25 years and revealed an increase in the CSA of the quadriceps, hamstrings, and adductor muscles (+7%–10%).

Regarding training frequency, our findings indicate that the effects of plyometric jump training on skeletal muscle hypertrophy were moderated by the weekly session frequency [β = 0.3233 (95% CIs = 0.2040–0.4425)] with a higher weekly session frequency inducing larger hypertrophic gains. Of note, most of the included studies used biweekly or triweekly plyometric jump training sessions. Specifically, three sessions of plyometric jump training per week showed large increases in skeletal muscle hypertrophy (12.8%–25.8% increase in vastus lateralis CSA) (Earp et al., 2015) compared with smaller gains following two weekly sessions (14% increase in thigh muscle volume) (Fathi et al., 2019). This is in agreement with the literature about traditional resistance training (Wernbom et al., 2007; Schoenfeld et al., 2019; Schoenfeld et al., 2021). More specifically, in a systematic review with meta-analysis of the effects of traditional resistance training frequency on skeletal muscle hypertrophy in healthy individuals, Schoenfeld et al. (2019) reported a slightly larger effect of higher compared with lower frequencies of training on hypertrophic outcomes when training volume was not equated between conditions. However, under equated-volume conditions, no additional effects of higher compared with lower frequencies of training were reported (Schoenfeld et al., 2019). The same conclusion was made in a recent consensus review in that a higher number of traditional resistance training sessions per week (e.g., 3 sessions vs. 1 session) is recommended to gain more muscle mass (Schoenfeld et al., 2021). The same authors attributed the larger benefits of manipulating training frequency to its potential effect on the distribution of the weekly training volume (Schoenfeld et al., 2021). Future studies should endeavor to better understand the interaction between plyometric jump training frequency and hypertrophic adaptations, particularly in context with alterations in volume and intensity.

With respect to age, the positive point estimate suggests that older participants tend to achieve larger hypertrophic adaptations (Figure 5; https://osf.io/g8aet/). The same observation was noted for total training period (Figure 5; https://osf.io/g8aet/), with longer exposure to plyometric jump training appearing to induce larger hypertrophic gains. However, for a single session duration (Figure 5; https://osf.io/g8aet/), there is a tendency for shorter sessions to induce larger hypertrophic gains. It should be noted though that these observations are not conclusive and hence need to be confirmed in future studies.

### 4.3 Limitations

Some limitations of this meta-analysis need to be acknowledged. The lack of a standardised method to assess skeletal muscle hypertrophy can constitute a limitation given that different assessment techniques may disagree with one another (e.g., macroscopic vs. microscopic) (Haun et al., 2019). Indeed, studies included in this analysis have used a wide range of methods to assess skeletal muscle hypertrophy. More specifically, the included studies relied upon macroscopic assessment methods (e.g., B-mode ultrasound) (Skurvydas and Brazaitis, 2010; Fouré et al., 2011; Fouré et al., 2012; Correa et al., 2012; Earp et al., 2015; Allison et al., 2018; McKinlay et al., 2018), microscopic methods (e.g., biopsy) (Kyröläinen et al., 2005), and a prediction equation to assess muscle volume and CSA (Chelly et al., 2010; Chelly et al., 2014; Chelly et al., 2015; Fathi et al., 2019). In fact, the prediction equation represents an indirect tool that provides a crude estimate of skeletal muscle hypertrophy and does not allow for the differentiation between muscle tissue, fat tissue, and bone (Chelly et al., 2006; Grgic et al., 2019). Additionally, although skeletal muscle hypertrophy was a primary outcome in most of the included studies (Kyröläinen et al., 2005; Marković et al., 2005; Herrero et al., 2006; Correa et al., 2012; Chelly et al., 2014; Chelly et al., 2015; Earp et al., 2015; Allison et al., 2018), it was in some other studies (Chelly et al., 2010; Skurvydas and Brazaitis, 2010; Fouré et al., 2011; Fouré et al., 2012; McKinlay et al., 2018; Fathi et al., 2019; Cherni et al., 2020) a secondary outcome. As such, caution must be taken when interpreting the present findings. Furthermore, moderator analyses were computed independently, ignoring any potential interdependency (interaction) between variables. Therefore, the results of univariate analyses must be interpreted with caution. Finally, although we have included studies that used athletic samples, the resistance training expertise of these participants is generally not clear. Thus, results cannot necessarily be generalized to well-trained individuals. Further studies are warranted to determine the effects of plyometric jump training on skeletal muscle hypertrophy in those with significant resistance training experience.

## 5 Conclusion

Contrary to common belief, plyometric jump training seems to induce skeletal muscle hypertrophy, albeit to a small to moderate magnitude. Such an effect appears to be consistent across different ages, sexes, and training experiences. Furthermore, there is evidence of relatively larger hypertrophic adaptations in non-athletes compared with athletes, with no clear evidence that either age or sex moderated the effects of plyometric jump training on skeletal muscle hypertrophy. Regarding the assessment methods, it is advisable to favor the use of ultrasound and other validated site-specific imaging modalities over the prediction equation to provide more accurate insights into skeletal muscle hypertrophy. Moreover, meta-regression analyses suggest that the effects on skeletal muscle hypertrophy are moderated by the weekly session frequency with higher frequencies inducing larger gains in skeletal muscle hypertrophy. However, there is no clear evidence that total training period, single session duration, and the number of jumps per week moderated the effects of plyometric jump training on skeletal muscle hypertrophy.

## 6 Future Research Perspectives

Given that skeletal muscle hypertrophy was a secondary outcome in many of the included studies, future investigations of high methodological quality (e.g., randomized-controlled trials) where skeletal muscle hypertrophy is the primary endpoint are required to substantiate the present findings. In addition, the effects of plyometric jump training on muscle protein synthesis are still unknown. Therefore, future research should explore the mechanisms by which plyometric jump training induces skeletal muscle hypertrophy. That said, researchers’ attention should be redirected toward the effects of single-mode plyometric jump training on the anabolic signaling pathway. Such studies will provide novel insights into the mechanisms of plyometric jump training-related hypertrophic adaptations in humans. Further, there is a need for future longitudinal studies to compare and contrast the effects of different plyometric jump training volumes, frequencies, and intensities on skeletal muscle hypertrophy. Also, we were able to locate only three studies that included female participants (Skurvydas and Brazaitis, 2010; Correa et al., 2012; Cherni et al., 2020) and only two studies that included older adults (Correa et al., 2012; Allison et al., 2018). Therefore, future investigations should recruit females as well as older adults to fill this gap in the literature. Furthermore, the effects of plyometric jump training vs. traditional resistance training on skeletal muscle hypertrophy have never been meta-analyzed. Of note, there is only one previous review on the topic, but it is descriptive and included only six studies (Grgic et al., 2020), limiting the veracity of its main findings. As such, there is a need to aggregate data from the available literature to draw statistical inferences on the effects of plyometric jump training vs. traditional resistance training on skeletal muscle hypertrophy when there are a sufficient number of studies on the topic. Moreover, given that the combination between plyometric jump training and traditional resistance training favors an anabolic hormonal milieu (Beaven et al., 2011; Ali et al., 2019), it would be relevant to determine optimal combination strategies between plyometric jump training and traditional resistance training to maximize skeletal muscle hypertrophy.

## Data Availability

Publicly available datasets were analyzed in this study. This data can be found here: https://osf.io/bf478/.

## Footnotes

1 Training experience was determined with regard to the context from which participants were recruited. Athletes were recruited from specific sport settings (e.g., sports clubs or teams) and were actively participating in competitive events while non-athletes were recruited outside this setting [e.g., educational institutions (school, university) or rural/urban communities] and were not actively participating in sports competition (Araújo and Scharhag, 2016).
